# 55 Predictive factors of infection in patients with systemic autoimmune disease on biotherapy

**DOI:** 10.1093/rheumatology/keac496.051

**Published:** 2022-10-07

**Authors:** Boudokhane Manel, Ben Ayed Hiba, Ben Yaacoub Sarra, Bourguiba Rym, Teyeb Zeineb, Ayari Mariem, Jomni Taieb, Abdelaali Imen, Belakhal Syrine, Dougui Mohamed Hedi

## Abstract

**Introduction:**

The increasing use of biologics in chronic inflammatory diseases is accompanied by an increased risk of infectious complications. Our objective was to determine the predictive factors for the occurrence of infection in patients on biotherapy.

**Methods:**

We conducted a single-center retrospective study in an internal medicine department over a period of 14-year [2009–2022], including the records of patients treated with a biologic agent presenting at least one infectious episode.

**Results:**

During the study period, 20 patients among 50 treated with biologic agent had at least one infection episode. The patient mean age was 50.8 ± 12.1 [24–76] years and the male to female ratio was 1:2. The mean age at biotherapy first use was 43 ± 12.1 [17–68] years. Infections occurred after a mean duration of 12 months [0.5–36] after the initiation of biotherapy. Eleven patients were followed for chronic inflammatory bowel disease, three patients for ankylosing spondylitis, three patients for vasculitis, two patients for rheumatoid arthritis and one patient for systemic scleroderma. Only three patients were diabetic and two had chronic pulmonary disease. Thirteen patients (65%) were treated by TNFα inhibitors and 35% with anti CD20 (Rituximab). Among TNFα inhibitors, seven infections occurred in patient treated with infliximab (41%), six with adalimumab (35%) and four with etanercept (24%). At the time of infection, half of the patients were treated by corticosteroid with a median dose of 32 mg/d [5–60] and a median duration of 11 months [2–90], while 40% of the patients were on conventional immunosuppressive therapy. The infection was severe, requiring hospitalization in eight cases (40%). A statistically significant correlation was found between the occurrence of infection and a duration of anti-TNFα treatment <6 months (*p* = 0.003) and history of crohn's disease (*p* = 004). Age >50 years at the time of biotherapy introduction did not correlate with a higher risk of infection (*p* = 0.7)

**Conclusion:**

Screening for infections in patient treated with biologic agents must be systematic, especially at the treatment initiation, in order to ensure better compliance and efficacy of the biotherapy.

